# Only Female Age, and Not Blood Type, Is Associated with Ovarian Reserve

**Published:** 2014-07-08

**Authors:** Özlem Şengül, Berna Dilbaz, Neslihan Yerebasmaz, Suat Dede, Şadıman Altınbaş, Salim Erkaya

**Affiliations:** Etlik Zübeyde Hanım Women’s Health Education and Research Hospital, Ankara, Turkey

**Keywords:** Blood Types, Ovarian Reserve, Age

## Abstract

**Background:**

The association between blood types and ovarian reserve is investigated in this study.

**Materials and Methods:**

As an index of ovarian reserve, women with a follicle stimulat- ing hormone (FSH) level of ≥10 mIU/ml in the early follicular phase were designated as having diminished ovarian reserve. In this prospective study, early follicular phase serum FSH and estradiol levels and blood types were evaluated in 500 patients who were admitted to the Infertility Department of Ministry of Health Etlik Zübeyde Hanım Women’s Health Training and Research Hospital between January 2012 and June 2012. Women with serum FSH level <10 mIU/ml formed group I, and women with serum FSH ≥10 mIU/ml formed group II. The prevalence of blood types in each group and their associa- tion with ovarian reserve were analyzed.

**Results:**

Out of 500 patients, 438 women were in group I, while 62 women were in group II. There was no statistically significant difference among the two groups in terms of blood group proportions (p=0.69), this did not change after age adjustment (p=0.77). The presence of A antigen (in A and AB blood type) (p=0.91), the blood type O (p=0.70), and the blood type B (p=0.51) were not statistically related to ovarian reserve after age adjustment. There was also no statistically significant correlation between rhesus factor and ovarian reserve after age adjustment (p=0.83). The only factor that affected ovarian reserve was age of patients (p=0.006).

**Conclusion:**

Blood groups do not constitute a risk or protective factor for ovarian reserve. Therefore, blood groups do not have any predictive value in evaluating ovarian reserve.

## Introduction

Women are born with a fixed number of follicles in their ovaries that decline with age culminating in the menopause at 50-51 years (1). As a result of the decrease in follicles, it has been shown that fertility starts to decline after the age of 30 leading to sterility at the age of 41 (2, 3). Socioeconomic changes have caused an increasing number of women delaying childbirth to later ages contributing to increased incidence of subfertility. Generally, age of the woman has a major predictive value in evaluating ovarian reserve (4). However, normal ovarian failure leading to natural menopause occurs with a wide range of age variation. Numerous tests have been used for evaluation of the ovarian reserve in order to predict the reproductive capacity (2). Among these ovarian reserve tests, anti-Mullerian hormone (AMH) and antral follicle count, early follicular phase serum follicle stimulating hormone (FSH) level appears to be the most commonly used test for detection of ovarian reserve, although contraversial findings have been reported (5). However, age still remains as having the highest predictive value.

A serum FSH level ≥10 mIU/ml is usually considered as a threshold for diminished ovarian reserve (6). There was no known association between blood groups and infertility. But, blood group A was reported to be associated with early onset ovarian hyperstimulation syndrome (7). In contrast, Belver et al. (8) found no relationship between early onset ovarian hyperstimulation syndrome and blood types. Nejat et al. (9) suggested that blood group A antigen was protective for ovarian reserve, while O antigen was a risk factor for diminished ovarian reserve independent of age. Our aim in this study is to clarify whether there exists an association between blood types and ovarian reserve.

## Materials and Methods

Five hundred women in the follicular phase of menstrual cycle between 18 and 45 years of age who applied to Infertility Department of Ministry of Health Etlik Zübeyde Hanım Women’s Health Training and Research Hospital between January 2012 and June 2012 were enrolled in this prospective study. The patients were either nulligravid admitted to the hospital for any gynaecologic reason or seeking infertility treatment. The demographic characteristics of the patients were obtained from the history of the patients. Serum FSH (mIU/ml) and estradiol levels (E2, pg/ml) were measured on the third day of the menstrual cycle and blood type (A, B, AB or O) was also determined. FSH ≥10 mIU/ml was accepted as a threshold for diminished ovarian reserve (6). Women with FSH <10 mIU/ml formed group I (n=438), and women whose FSH ≥10 mIU/ml formed Group II (n=62). The association between ovarian reserve and the blood types was investigated. The blood types and ovarian reserve were collated after adjustment of the age.

### Statistical Analysis

In independent groups, the difference between the two groups’ means was evaluated by t test (two tailed). The correlation between qualitative variables was evaluated by chi-square test and extension of Fisher’s exact test. Analysis after adjustment of age was done by multiple logistic regression analysis. Numerical data was presented as mean ± standard deviation (SD). A p value of <0.05 was accepted as statistically significant. Data analysis was performed using a Statistical Package for the Social Sciences (SPSS; SPSS Inc., Chicago, IL, USA) version 16.

### Ethical Considerations

The study was approved by the ethics board of the hospital. Informed consent was obtained from all the cases enrolled in the study.

## Results

There were 438 women (87.6%) in group I and 62 women (12.4%) in group II. A total of 447 of the 500 patients (89.4%) were seeking infertility treatment. The age of the patients in group I was statistically younger than group II (26.97 ± 5.60 years vs. 29.82 ± 7.69 years; p=0.006). There was no statistically difference between the two groups in terms of body mass indices (BMI), significantly (25.03 ± 4.45 kg/m^2^ vs. 24.73 ± 4.43 kg/m^2^; p=0.62). The demographical characteristics of the patients are shown in table 1. There was no statistically significant difference in terms of history of the patients between the two groups (p=0.93) in terms of previous ovarian or uterine surgery, hypothyroidism and other chronic diseases like diabetes mellitus, hypertension, epilepsy, migraine, etc. There was also no statistically significant difference between the two groups in parity, smoking, contraception, infertility, menstrual pattern and history of ovulation induction (p>0.05). The distribution of blood groups was: A (39.8%), B (15.4%), AB (9.4%), and O (35.4%). The distribution of blood types according to ovarian reserve is shown in table 2. There was no statistically significant difference among the two groups in terms of blood group proportions, and there was no correlation between ABO blood types and ovarian reserve (p=0.69), this did not change after age adjustment (p=0.77). Also, the blood type A (p=0.36) (p=0.58 after adjustment of age), the presence of A antigen (in A and AB blood type) (p=0.69) (p=0.91 after adjustment of age), the blood type O (p=0.98) (p=0.70 after adjustment of age), and the blood type B (p=0.56) (p=0.51 after adjustment of age) did not statistically significantly affected ovarian reserve. There was also no statistically significant correlation between rhesus factor and ovarian reserve (p=0.70), and this result persists after adjustment of the age (p=0.83). The only factor that affect ovarian reserve was age of the patients (p=0.006).

**Table 1:** The demographical characteristics of the patients


	FSH < 10 IU/ml	FSH ≥ 10 IU/ml	P value

**Age (Y)**	26.97 ± 5.60	29.82 ± 7.69	<0.05
**Body mass index (kg/m^2^)**	25.03 ± 4.45	24.73 ± 4.43	>0.05
**Gravida**	0.64 ± 0.93	0.94 ± 1.42	<0.05
**Parity**	0.38 ± 0.64	0.52 ± 0.78	>0.05
**Estradiol (pg/ml)**	50.27 ± 45.29	49.85 ± 54.48	>0.05
**Previous ovarian surgery**	10 patients	1 patient	>0.05
**Previous uterine surgery**	37 patients	7 patients	>0.05
**Hypothyroidism**	15 patients	2 patients	>0.05


FSH; Follicle stimulating hormone.

**Table 2:** The distribution of blood types according to ovarian reserve


Blood type	FSH <10 IU/ml	FSH ≥10 IU/ml	P value	Total

**A**	171	28	0.58	199
**B**	69	8	0.51	77
**AB**	43	4	0.34	47
**O**	155	22	0.70	177
**A Antigen positivity**	214	32	0.91	2.46
**Rhesus factor**	396	57	0.83	453


FSH; Follicle stinulating hormone.

## Discussion

According to the distribution of blood types in Turkey, 42.5% of the population have type A, 33.7% have type O, 15.8% have type B, and 8.0% have type AB (10). This distribution is close to the distribution in this study. Our findings demonstrate that blood groups are not related to ovarian reserve after age adjustment. This is conflicting to the data presented by Nejat et al. (9), as his findings suggested that while blood group A antigen was protective for ovarian reserve, blood group O constituted a risk factor for diminished ovarian reserve. However, in his study, timing of blood sampling was not valid, although the patients with estradiol levels more than 80 pg/ml were excluded from the study. Since FSH has great variations during menstrual cycle, the lack of exact timing may affect the results. In our study, all of the blood samples for measuring FSH and estradiol were obtained on the third day of the menstrual cycle. The factors that may affect ovarian reserve like smoking, body mass index and previous ovarian surgery were also taken into consideration and there was no statistically significant difference between the two groups with regard to the prevalence of these mentioned factors.

In previous work on a large cohort of women of multiple ethnic groups, it was found that there was a significant difference between races in terms of ovarian reserve since AMH and inhibin B levels were signficantly lower in the African American women (11). There was no potential race effect in our results since all of our patients were Caucasian women. Moreover, de Mouzon et al. (12) could not find an association between blood types and ovarian reserve using AMH for evaluation of ovarian reserve. Although AMH may have a higher predictive value for evaluation of ovarian reserve (13), early follicular phase FSH level is commonly used in routine clinical evaluation of the infertile women. Our results are similar to those of de Mouzon et al. (12), as early follicular phase serum FSH levels were used in this study and no correlation was found between ovarian reserve and rhesus factor.

Our results have shown that, blood groups do not constitute a risk or protective factor for ovarian reserve. Therefore, blood groups should not be taken into account while evaluating ovarian reserve. However, our results are needed to be validated by further studies.

## Conclusion

Blood groups do not constitute a risk or protective factor for ovarian reserve. Therefore, blood groups do not have any predictive value in evaluating ovarian reserve.
